# On Epilepsy

**Published:** 1860-04-01

**Authors:** 


### Art. IX.—ON EPILEPSY.

Two valuable contributions have recently been made to the English literature of epilepsy and disorders akin to epilepsy, by the translation for the New Sydenham Society of the undermentioned essays of Professor Schroeder van der Kolk, and Drs. Kussmaul and Tenner.*

The starting-point and source of spasm in epilepsy, according to Professor van der Kolk, is to be found in exalted sensibility and activity of the ganglionic cell of the medulla oblongata; and the grounds upon which this opinion is based are to be found in the seat of the characteristic spasms, in the bilateral character of these spasms, and in the appearances disclosed after death.

The spasms of epilepsy begin in muscles, the nerves of which arise in the medulla oblongata—in muscles, that is to say, which are supplied by the facial, accessory, hypoglossal, and the portio minor trigemini; and in slighter cases, the spasms may be limited to the sphere of these nerves. The spasmodic contraction of the muscles of the chest, which is the most prominent and characteristic feature in the complete attack of epilepsy, is also supposed to point to the medulla oblongata as the origin of the evil, for a similar state of things is produced by the action of a strong stimulus upon the great afferent nerve of the medulla oblongata—the pneumogastric.

The bilateral character of the spasms is used as another argument in favour of this idea that the medulla oblongata is espe-

* 1. *On the Prominent Cause and Rational Treatment of Epilepsy.* By Professor Schroeder van der Kolk. Transl. by Dr. W. D. Moore, for the New Sydenham Society. 1859. 2. *On Epileptiform Convulsions from Hæmorrhage.* By Drs. Kussmaul and Tenner. Transl. by Dr. Browne, for the New Sydenham Society. 1859.

cially affected in epilepsy. The lateral halves of the medulla oblongata are connected in the most intimate manner by transverse fibres and commissures, and this intimate connexion between the two lateral halves is one principal point of difference between the medulla oblongata and the brain and spinal cord. So intimate, indeed, is the connexion between the two halves of the medulla oblongata that unilateral action is impossible. Moreover, it is evident in none of the natural movements of the features, or tongue, or voice, or breathing—movements more or less under the control of the medulla oblongata unless the muscles and nerves of the two sides concerned in these actions should act in concert. In the brain and spinal cord, on the other hand, where there is no such intimate anatomical and functional connexion between the two lateral halves, one side may act or fail to act independently of the other side. Hemiplegia is an illustration of such failure of action. Now in epilepsy, though the spasms may undoubtedly affect one side more than the other side, they are always more or less *bilateral,* and for this reason Professor S. van der Kolk supposes them to spring more or less from the medulla oblongata.

The appearances after death also point especially to the medulla oblongata. In early cases we may often fail to find any characteristic changes; but in confirmed cases, Professor Schroeder van der Kolk finds unequivocal changes in the medulla oblongata. Thus he finds this organ often harder than natural from the interstitial deposit of a minutely granular albuminous matter, or else softened, swollen, and exhibiting signs of fatty degeneration. He also finds very marked changes in the bloodvessels, and to these he directs particular attention. In fifteen cases the posterior half of the medulla oblongata, from the fourth ventricle, was redder and more hyperæmic than in the natural state, and this whether death happened in an attack or not; and on minute examination, the bloodvessels in the red and congested portions were found to be dilated to thrice their natural dimensions, and with their walls much thickened. On comparing, moreover, the medulla oblongata of epileptics who bit their tongue with the medulla oblongata of epileptics who did not bite their tongue, the dilatation of vessels was seen to be most marked in the course of the hypoglossus and the corpus olivare in the former case, and in the course of the vagus in the latter case.

The facts upon which Professor Schroeder van der Kolk founds his theory of *exalted action* of the medulla oblongata in epilepsy may be questioned. With him spasm is the great argument in favour of exalted action—an argument which will be disallowed by Dr. Radcliffe and those who think with him. A full bounding pulse, as implying an excited condition of the circulation, is also used as an argument in favour of exalted action in the medulla oblongata; but here again is a doubt, for it has been shown, we think conclusively, that a full bounding arterial pulse has nothing whatever to do with the phenomena of epilepsy. Nor do we think that the after arguments used are more cogent than these. We think, indeed, that Professor Schroeder van der Kolk does not establish his theory of *exalted action* of the medulla oblongata as the starting-point and source of spasm in epilepsy; but on the other hand, we think that he has given us additional reasons for looking to the medulla oblongata if we would hope to read aright the most characteristic symptoms of epilepsy.

The treatment recommended is in accordance with the notion that there is exalted action to be subdued—prudent diet, no stimulants, daily evacuations from the bowels, tartar emetic, digitalis, local bleedings high in the neck, especially with cupping glasses, setons, issues, actual cautery, also high in the neck, and so on. Zinc and nitrate of silver are thought to act beneficially in the cases where they do so act, by moderating intestinal irritability. Sulphate of copper is condemned. Worms, in particular, are to be sought for and removed.

The chief results arrived at by Drs. Kussmaul and Tenner in their essay may be noted in the following general summary:—

1. The convulsions appearing in profuse hæmorrhage of warm-blooded animals (including man) resemble those observed in epilepsy.

2. When the brain is suddenly deprived of its red blood, convulsions ensue of the same description as those occurring subsequent to ligature of the great arteries of the neck.

3. Epileptic convulsions are likewise brought on when the arterial blood rapidly assumes a venous character, as, for example, when a ligature is applied to the trachea.

4. It is highly probable that in these cases the attack of spasms depends upon the suddenly interrupted nutrition of the brain. It is not caused by the altered pressure which the brain undergoes.

5. Epileptic convulsions in hæmorrhage do not proceed from the spinal cord.

6. Neither do they proceed from the cerebrum.

7. Their central seat is to be sought for in the excitable districts of the brain lying behind the thalami optici.

8. Anæmia of those parts of the brain situated before the crura cerebri produces unconsciousness, insensibility, and paralysis in human beings; if spasms occur with these symptoms, some excitable parts behind the thalami optici must have likewise undergone some change.

9. Anæmia of the spinal cord produces paralysis of the limbs, of the muscles of the trunk, and of respiration. When the anæmia suddenly attains its greatest intensity, then only, and even then but rarely, do slight trembling movements of the limbs precede paralysis. The sphincter ani acts analogously to the constrictor muscles of the face in anæmia of the brain, that is, it contracts spasmodically before it relaxes.

10. Convulsions from hæmorrhage are neither physical nor reflective.

11. Convulsions from hæmorrhage do not ensue—

*a.* In cold-blooded animals, at least not in the frog.

*b.* When the hæmorrhage is slow, so that the vital power is only gradually consumed.

*c.* When the animals are very much debilitated.

*d.* When the nutrition of the spinal cord has suffered.

*e.* When large pieces of the excitable districts of the brain have been removed.

*f.* In animals subjected to etherization.

*g.* Doubtless also when excitable districts of the brain have undergone certain pathological alterations.

12. As suffocation brings on convulsions, and etherization averts them, it is evident that etherization and asphyxia are two different conditions.

13. The brain of warm-blooded animals can only be deprived of red blood for a short time; otherwise it loses its capability of resuming its functions when again supplied with the nutritive fluid, and the appearance of death becomes a reality. The brain of some rabbits preserved this capability for two minutes.

14. It is sometimes observed, after the arteries of the neck have been tied, that the muscles of the trunk perish and take on the rigor mortis before the action of the left heart is extinct. Hence the left heart is not always the *primum moriens* among the muscular organs.

15. Contraction and subsequent extreme dilation of the pupils in the agonies of death, is no certain sign of real death and of the incapability of being revived, as maintained by Bouchut.

16. To cure epileptic attacks caused by anæmia, there is no better method than that of renewing the supply of red blood.

17. The debilitating method of treating epilepsy, especially by *abstracting* blood, should almost always be rejected.

18. The quantity of blood in the cranial cavity can by way of experiment on the living subject be considerably increased or diminished.

19. Hyperæmia in the cranial cavity is caused by releasing the stoppage of circulation in the cervical arteries (arterial congestion), by tying the cervical veins (venous congestion), especially by simultaneously dividing the cervical branches of the sympathetic nerve (venous arterial congestion), and lastly by tying the trachea during inspiration (venous congestion by asphyxia).

20. Anæmia in the cranial cavity is produced by hæmorrhage and by tying the cervical arteries (passive anæmia), as well as by electric excitation of the vaso-motor nerves of the head (active anæmia).

21. The quantity of blood contained in the cranial cavity after the application of a ligature to the arteries is greater than after hæmorrhage; the anæmia as regards small arteries, the capillaries, and the smallest veins being always present to a greater extent.

22. From the quantity of blood contained in the skull after death, it is seldom possible to draw certain conclusions with respect to the quantity contained during life. The death-struggle brings on numerous conditions altering the circulation of the blood in the skull, and even in the corpse the quantity of blood may still undergo alterations.

23. The phenomena of the incomplete epileptic attack can be explained by alterations occurring in the cerebrum only; whilst the phenomena of the complete attack presuppose an alteration of the whole brain. Convulsions in epilepsy are justly styled cerebral ones, and the spinal cord probably plays only the part of a conductor, transferring the impetuses it receives from the brain to the muscles.

24. Circumscribed anatomical alterations of the brain or alterations of protracted duration cannot be regarded as the proximate cause of epileptic attacks, but may cause epileptic *affections* (dispose to epilepsy).

25. Pathological anatomy cannot give any explanation as to the nature of epilepsy.

26. Suddenly withheld nutrition is only one of the causes by which the brain is brought into that peculiar internal condition which is manifested in the form of an epileptic attack.

27. Arterial congestion of the brain, does not seem to be capable of producing any other symptoms than those of paralysis (dizziness and apoplexy).

28. Venous congestion of the brain, as well as arterio-venous congestion, brings about conditions which belong more to those of apoplexy than to those of epilepsy, and are characterized by paralysis of the glottis, together with a slower respiration and slight spasmodic symptoms.

29. Marshall Hall's sphagiasmus and trachelismus are not to be regarded as a source of epileptic attacks, but laryngismus will produce them. All theories are false which assert the epileptic attack to be derived from a sudden determination of blood, whether active, passive, or mixed.

30. It is probable that certain forms of epilepsy result from a spasm of the muscular coats of the cerebral arteries.

31. The epileptic affection, which disposes to the attacks, occupies either the whole of the brain, or some districts only, and by it the brain is brought into that altered state on which the epileptic attack is based.

32. The medulla oblongata, as being the part whence the nerves causing the constriction of the glottis and the vaso-motor nerves take their rise, seems frequently to be the spot from which eclamptic and epileptic attacks proceed.

All who read the essays that we have thus briefly examined will feel satisfied that the council of the New Sydenham Society have acted most wisely in presenting them to their members, and will feel under an obligation to Drs. Moore and Browne for the able manner in which the translations have been effected.

---

